# The Nutritional Status of Patients with Heart Failure and Its Impact on Patient’ Outcomes—The Center’s Own Experience

**DOI:** 10.3390/nu17050761

**Published:** 2025-02-21

**Authors:** Natalia Świątoniowska-Lonc, Marek Aureliusz Mak, Filip Klausa, Krzysztof Ściborski, Waldemar Banasiak, Adrian Doroszko

**Affiliations:** 1 Department of Cardiology, Center for Heart Diseases, 4th Military Hospital, 50-981 Wroclaw, Poland; k.sciborski@op.pl (K.Ś.); banasiak@4wsk.pl (W.B.); adrian.doroszko@pwr.edu.pl (A.D.); 2 Department of Cardiac Surgery, Center for Heart Diseases, 4th Military Hospital, 50-981 Wroclaw, Poland; aureliuszm@prokonto.pl (M.A.M.); filip.klausa@gmail.com (F.K.); 3 Clinical Department of Cardiology, Faculty of Medicine, Wroclaw University of Science and Technology, 50-370 Wroclaw, Poland

**Keywords:** nutritional status, heart failure, mortality, length of stay

## Abstract

**Background**. The nutritional status of patients in hospitals has a significant impact on the effectiveness of treatment, the occurrence of complications, and the length of hospitalization. **The purpose of this study** was to evaluate the nutritional status of patients with heart failure (HF) and its impact on patient outcomes. **Material and Methods**. This study included 213 patients (153 men, 71.8%) aged 74.7 ± 14.3 years treated for HF at the cardiology clinic of the 4th Military Clinical Hospital between 2018 and 2021. Sociodemographics, clinical data, the Model for End-Stage Liver Disease (MELD), CHILD-PUGH, and the Nutritional Risk Score (NRS) were analyzed. **Results**. Patients at high nutritional risk (NRS ≥ 3 score) were older (85 years vs. 75 years; *p* < 0.001), had longer hospitalizations (12 days vs. 9 days, *p* = 0.027), lower hemoglobin (10.5 g/dL vs. 11.7 g/dL, *p* = 0.001), lower TIBC (292 vs. 336; *p* = 0.012), and iron (32 mg/nL vs. 39 ng/mL, *p* = 0.009) compared with patients at low risk (NRS < 3 score). Patients hospitalized ≤7 days had significantly lower CHILD-PUGH score compared with patients hospitalized >7 days. Patients hospitalized ≥14 days were significantly more likely to die compared with other groups of HF patients (10.6% vs. 0.0%, *p* = 0.004). **Conclusions**. Abnormal nutritional status among hospitalized HF patients is associated with longer hospitalization duration and higher rates of death. In addition to clinical factors, the CHILD-PUGH scale can be helpful in estimating the length of hospitalization of HF patients. It is necessary to determine the impact of nutritional status on the outcome of patients with heart failure in further multicenter prospective or interventional studies.

## 1. Background

Heart failure (HF) is a major global health problem, characterized by chronic progression and frequent exacerbations that significantly affect patient outcomes and the burden on healthcare systems [1]. An estimated 64.3 million people are living with heart failure worldwide [2]. In developed countries, the prevalence of diagnosed heart failure is generally estimated at 1% to 2% of the general adult population [3,4,5,6]. Based on the latest data compiled by the Polish Ministry of Health, it is known [7] that the number of patients diagnosed with HF in Poland increased to 1.02 million between 2014 and 2019. In addition, there was a 26% decrease in the number of patients with a de novo diagnosis of HF in 2019 compared to 2014. The apparent decrease in the number of new cases and total prevalence in 2020–2021 is most likely a result of the COVID-19 pandemic and associated impaired access to healthcare, including primary diagnosis [8]. The total number of heart failure patients in Poland was 2413 per 100,000 population in 2021 [7].

The growing public health burden of cardiovascular disease (CVD) is a global challenge, particularly in the context of dietary and lifestyle changes [9]. The rising prevalence of hypertension, obesity, and diabetes, which are key CVD risk factors, is closely linked to the prevalence of a diet low in fiber and rich in processed foods and saturated fat [9]. In addition, decreased physical activity and sedentary lifestyles further increase the risk of developing these diseases [10]. Low- and middle-income countries, where urbanization encourages unhealthy habits, are particularly vulnerable [11]. Studies indicate that lifestyle changes, including increased fiber intake and regular physical activity, can significantly reduce the incidence of CVD [12,13].

The Mediterranean diet has long been recognized as a dietary model beneficial to cardiovascular health [14]. However, studies indicate a decline in its adherence, which may undermine its original benefits. The traditional Mediterranean diet, rich in fruits, vegetables, nuts, and olive oil, is associated with a lower risk of cardiovascular disease [15]. However, modern changes in eating habits, such as the increase in consumption of processed foods and saturated fats, are leading to a shift away from this model. Additionally, barriers to adherence to the Mediterranean diet in non-Mediterranean populations may affect its effectiveness in cardiovascular disease prevention [16]. As such, promotion and education about traditional dietary patterns are key to maintaining its health-promoting properties.

The European Society of Cardiology (ESC) guidelines [17] emphasize the role of non-pharmacological management in the management of heart failure. Available studies show a relationship between adherence to non-pharmacologic recommendations and outcomes for patients with heart failure.

Nutritional status is a modifiable risk factor in the management of chronic diseases, including HF [18]. Despite advances in pharmacological and interventional treatments, malnutrition remains a common and underrecognized problem in patients with HF. Studies indicate that poor nutritional status in this group is associated with higher rates of hospitalization, prolonged length of hospital stay, and increased mortality [19,20,21]. In addition, the interaction between HF and nutritional deficiencies is complex, involving low-grade inflammation, gastrointestinal congestion, and metabolic disorders [22]. These factors can lead to a paradoxical condition in which obese patients simultaneously exhibit micronutrient deficiencies, further complicating their treatment [23]. Patients hospitalized for HF exacerbations often present complex clinical profiles in which comorbidities and nutritional status play an important role [24,25]. In this context, malnutrition and related risk factors become key determinants of recovery, length of hospitalization, and mortality.

The purpose of this study is to assess the nutritional status of patients hospitalized for exacerbations of HF and its impact on clinical outcomes, such as length of hospital stay and in-hospital mortality. The analysis focuses on a cohort of patients treated at a single center, using standardized nutritional and clinical assessment tools. By examining the relationship between nutritional parameters, clinical characteristics and patient outcomes, this study aims to highlight the importance of early nutritional screening and individualized interventions in the management of HF.

## 2. Material and Methods

This retrospective study analyzed the data of 213 patients hospitalized between 2018 and 2021 for heart failure exacerbations at the cardiology clinic of the 4th Military Clinical Hospital. The study was conducted in accordance with the Strengthening the Reporting of Observational Studies in Epidemiology guidelines.

Inclusion criteria for the analysis were age ≥ 18 years, diagnosis of heart failure (ICD10: I50.9), complete laboratory data (whole blood count, CRP, ALT, AST, creatinine, ferrum, albumin, bilirubin, PT/INR, %SATTF, TIBC, APTT) and hospitalization in the cardiology clinic between 2018 and 2021.

The standardized research instruments used in the study are shown in Table 1.

In addition to standardized clinical scales, information was collected on age, sex, height, weight, BMI, laboratory parameters (bilirubin, creatinine, albumin, INR, %SATTF, iron, ferritin, TIBC, APTT, blood count, CRP, ALT, ASP), the length of hospitalization, and the mode of discharge.

Clear definitions of all terms and variables were established at the hospital, and detailed training was conducted for all those involved in data collection. Standard forms were used. Electronic forms with built-in consistency checks were used to minimize errors, statistical methods were used to identify anomalies and potential errors in the data, such as outlier analysis.

The study was conducted according to the guidelines of the Declaration of Helsinki and approved by the Bioethics Commission at the Military Medical Chamber (decision number 213/2022, approved on 22 April 2022).

The primary endpoints were treatment efficacy (cured), incidence of complications (ascites), and length of hospitalization in two groups of patients at nutritional risk (according to NRS 2002). The G*Power (v. 3.1.9.7) program was used to estimate the minimum sample size. Assuming a significance level of α = 0.05, a power of test 1 − β = 0.80, and an effect size of Es = 0.30 (medium effect), the total sample size was *N* = 64. The study group of patients was increased to 213, keeping in mind the secondary end effects. Post hoc test power calculations were also performed. For the two-sided Mann–Whitney test (two sides) for the observed difference in hospitalization time of three days, the effect size was Es = 0.66. Assuming a significance level of α = 0.05 and sample sizes: N1 = 181, N2 = 32, the power of the test was 1 − β = 0.919.

The analysis was performed in R software, version 4.4.2 [29]. Distributions of quantitative variables were presented by means, standard deviations, medians and quartiles, while distributions of qualitative variables were presented by number and percentage of occurrences for each of their values. The chi-square test and Fisher’s exact test were used to compare the qualitative variables of the groups. The Mann–Whitney test was used to compare the quantitative variables of two groups, while the Kruskal–Wallis test (followed by the post hoc Dunn test) was used for comparisons between more than two groups. In all cases, Holm–Bonferroni correction for multiple comparisons was applied. A significance level of *p* < 0.05 was adopted for all statistical tests.

## 3. Results

This study analyzed a cohort of 213 patients (including 153 men, 71.8%) aged 76 [66; 85] years and treated for heart failure at the cardiology clinic of the 4th Military Clinical Hospital between 2018 and 2021. Clinical data are presented in Table 2.

### 3.1. Nutritional Assessment

In a group of 213 patients, the level of nutritional status expressed on the NRS scale was between 0 and 7 points (median Me = 1 point).

Table 3 shows the characteristics of 213 patients in groups differing in malnutrition risk. The risk of malnutrition expressed by the Nutritional Risk Screening (NRS)-2002 scale ≥ 3 affected 15.0% of the subjects.

- Cured: NRS < 3 vs. NRS ≥ 3 (95.0% vs. 84.4%, *p* = 0.026),
- Death: NRS < 3 vs. NRS ≥ 3 (2.2% vs. 12.5%, *p* = 0.005).

Patients at high nutritional risk (NRS ≥ 3 score) were older than patients at low risk (NRS < 3 score) by an average of 10 years (85 years vs. 75 years; *p* < 0.001), were shorter by 3cm (167 cm vs. 170 cm, *p* = 0.005), and lighter by 5 kg (75 kg vs. 80 kg, *p* = 0.007). Hospitalization time was longer by an average of 3 days (12 days vs. 9 days, *p* = 0.027), hemoglobin levels were lower by an average of 1.2 g/dL (10.5 g/dL vs. 11.7 g/dL, *p* = 0.001), iron levels were lower by 7 ng/mL (32 mg/nL vs. 39 ng/mL, *p* = 0.009), and TIBCs were lower compared with the low nutritional risk group (292 vs. 336; *p* = 0.012). 

### 3.2. Length of Stay in Hospital 

In the group of 213 patients, the duration of hospitalization ranged from 2 to 102 days (median Me = 10 days). Patients were classified into one of three groups: I—duration of hospitalization up to 7 days, II—duration of hospitalization from 8 to 13 days, III—duration of hospitalization of 14 days and longer.

Patients hospitalized <7 days had significantly lower body weight, lower BMI, lower CRP, creatinine, a lower CHILD-PUGH score, and higher albumin, iron, ferritin, and TIBC levels compared with patients hospitalized >7 days (Table 4). Patients hospitalized ≥14 days were significantly more likely to die compared with other HF patient groups (10.6% vs. 1.4% vs. 0.0%, *p* = 0.004). Patients hospitalized for 8 to 13 days were significantly more likely to have completed treatment compared to other groups of HF patients (respectively: 97.3% vs. 97.2% (≤7 days) vs. 84.9% (≥14 days); *p* = 0.009).

### 3.3. Effect of Treatment 

In a group of 213 patients, 199 patients were cured (group A), 6 patients were transferred to another hospital (group B), and 8 patients died in the hospital (group C). The results of the comparisons of the analyzed parameters are shown in Table 5.

The NRS in group C (i.e., those patients who died in hospital) was significantly higher compared with patients in group A who completed the therapeutic process (1 point vs. 2.5 points; *p* = 0.005).

The duration of hospitalization in the group of patients who died was significantly longer compared with that of patients who completed the therapeutic process (9 days. vs. 25 days; *p* = 0.002). 

Statistically significant differences between groups A, B, and C were also observed in terms of blood ALT enzyme levels and albumin levels. ALT levels in group B (patients referred for further treatment at another hospital or for outpatient treatment) were significantly higher compared with patients in group C (those who died in hospital) by an average of 20 U/L (34 U/L vs. 14 U/L, *p* = 0.015). The albumin concentration in group A (patients who completed treatment) was significantly higher compared with patients in group C (who died in hospital) by an average of 0.53 g/dL (3.70 g/dL vs. 3.17 g/dL, *p* < 0.001).

## 4. Discussion

This study aimed to assess the nutritional status of patients hospitalized for exacerbations of HF and its impact on clinical outcomes such as length of hospital stay and in-hospital mortality. The analysis included a cohort of patients treated at a single center, using standardized nutritional and clinical assessment tools. By examining the relationship between nutritional parameters, clinical characteristics, and patient outcomes, this study aimed to highlight the importance of early nutritional screening and individualized interventions in the treatment of HF.

Cardiovascular disease (CVD) is the leading cause of mortality worldwide [30]. CVD will remain the leading global cause of mortality, causing an estimated 23 million deaths by 2030 [31]. Preventive measures such as adopting a healthy lifestyle reduce the risk of myocardial infarction (MI) by 81 to 94% [32], while treatment with pharmacotherapy alone leads to a reduction of 20 to 30% [33]. Thus, nutrition is the most important behavioral factor in preventing premature death and disability from cardiovascular disease, ahead of abstinence from smoking and physical activity [34].

Behavioral factors are also associated with insulin resistance and diabetes, which significantly increases the risk of cardiovascular disease through accelerated atherosclerosis, endothelial dysfunction, and chronic inflammation [35]. Diabetes accelerates the progression of heart failure through insulin resistance, chronic inflammation, and oxidative stress, leading to myocardial damage [36]. Hyperglycemia promotes endothelial dysfunction, vascular stiffness, and cardiac remodeling, which worsens cardiac performance [37,38]. Metabolic disorders such as dyslipidemia and obesity exacerbate insulin resistance and oxidative stress, creating a vicious cycle between diabetes and heart failure [38]. The result is impaired cardiac contractility and increased sodium retention, increasing the risk of hospitalization and death [39]. It is noteworthy that excessive energy and fat intake increases the risk of obesity, but can also paradoxically go hand in hand with a deficiency of essential micronutrients [40]. Obesity seems to represent a new type of malnutrition as a result of unhealthy eating habits, poor absorption, and altered metabolism of micronutrients. Perhaps the responsible phenomenon is related to systemic chronic low-grade inflammation [22,41]. According to the World Health Organization, overweight and obese people often have an unrecognized risk of malnutrition [42]. Obesity is a condition of fat accumulation, and this is often paradoxically accompanied by quality deficiencies. This is the result of a diet lacking sufficient amounts of protein, vitamins, and minerals or conditions associated with excessive loss of these, and metabolic disorders. The risk of malnutrition is particularly higher in patients with a BMI > 30 kg/m^2^ [41] and is particularly exacerbated when weight loss occurs over 3 months (e.g., due to illness). About 50% of patients in the Polish population are malnourished. It has been shown that 38% of patients meet the criteria for malnutrition as assessed by the Nutritional Risk Screening 2002 (NRS) form [43] before planned surgery. Nearly half of these patients have a BMI above 25kg/m^2^.

In the Polish population setting, the risk of malnutrition is assessed only in the inpatient setting based on the mandatory NRS 2002 or Subjective Global Assessment (SGA) card assessment at patient admission, introduced in 2012. Clinical tools such as the Model for End-Stage Liver Disease (MELD) [44,45] and the CHILD-PUGH scale [11] are also widely used and can predict outcomes for hospitalized patients. The predictive properties of the MELD scale have been confirmed for the prognosis of patients with end-stage heart failure and liver disease awaiting heart transplantation [44]. In a study by Naghashzadeh et al. [46], the MELD was shown to be an effective predictor of perioperative risk stratification in patients with congestive hepatopathy and heart failure undergoing heart transplantation. The Child-Pugh score was not significantly associated with the prediction of mortality. In our study, a higher CHILD-PUGH score was associated with longer hospitalization for HF. Due to conflicting results in the literature, the use of the CHILD-PUGH and MELD scales in the HF patient population requires further study.

Data collected in the cardiology clinic on the basis of such cards indicate the necessity to consider pre-procedural nutritional treatment due to reductions in the length of hospitalization reducing the incidence of complications and reducing mortality. The n = 231 study group showed no statistical differences in gender in nutritional status. The mean BMI was estimated at 28.1 ± 6.2 kg/m^2^ and, therefore, on average, the study population was an overweight population. Malnutrition was assessed in this group according to the NRS2002 scale and averaged 1.2 ± 1.2 points. Approximately 13% of the patients evaluated had more than 3 points on this scale (n = 32) and these patients should be considered malnourished. They were mainly statistically significantly older patients on average by 10 years (85 years vs. 75 years; *p* < 0.001), they were 3 cm shorter than low-risk patients (NRS < 3 score) (167 cm vs. 170 cm, *p* = 0.005), and lighter by 5 kg (75 kg vs. 80 kg, *p* = 0.007), making the differences in BMI not statistically significant (27.4 [24.1; 31.3] vs. 25.8 [24.0; 28.5], *p* = 0.148).

In the context of the growing number of overweight and obese patients who may be malnourished, it is crucial to understand that BMI is not the only indicator of nutritional status, and that age and changes in body weight are important risk factors. In addition to the scales used, assessment of biochemical parameters is also used in risk assessment. In the study group, the assessment of plasma albumin levels, which is often used in the standard diagnostic panel on admission, was considered useless. The measurement of this indicator is controversial, because, in addition to nutritional status, it also reflects the severity of the disease and the hydration status of the body. However, regardless of this, most authors consider that a concentration of this protein below 3.5 mg/dL is indicative of malnutrition. It was statistically insignificant in both study groups.

The duration of hospitalization, which is highlighted in the literature as well as in our study, was prolonged by 25% (9 [6; 15] vs. 12 [9; 19] *p* = 0.027). Hospitalization was prolonged in malnourished patients by 5 to 6 days depending on the severity of the disease [47]. This is of great importance due to the need to implement nutritional support during hospitalization, which allows for lower mortality rates, fewer unplanned hospital readmissions, higher energy and protein intake, and greater weight gain than patients without nutritional support [23,24,48,49]. At the same time, reducing the length of hospitalization reduces the total cost of hospitalization, defined as the product of the number of bed days each patient spent on each type of unit and the cost per bed day. The bed-day cost includes medical care (e.g., drugs, parenteral, and enteral nutrition), procedures, nursing, and other services, such as dietary services, as well as meals, cleaning, and logistics. Depending on the funding system in each country, the cost of treating patients at nutritional risk was 46% higher than those not at risk [49].

In the malnourished group, lower hemoglobin levels and lower blood iron levels were observed. HF patients often suffer from malnutrition or cardiac cachexia, which is associated with loss of muscle mass and proteins, including albumin. Decreased albumin levels are a marker of malnutrition, which negatively affects the body’s recovery capacity, prolongs recovery time, and increases the risk of complications [50,51]. Albumin not only reflects nutritional status but also overall inflammation, severity of illness, and the body’s ability to adapt and recover. In our study, a shorter period of hospitalization was associated with lower body weight, lower BMI, CRP, creatinine, and higher albumin and iron levels compared with patients hospitalized >7 days. Patients hospitalized ≥14 days were significantly more likely to die compared with other groups of HF patients. Our observations are consistent with the available studies. In the Ancion et al. study [51], hemoglobin and serum albumin levels were found to be independent predictors of long-term mortality in a group of patients with acute heart failure. Similarly, in a study by Köseoğlu and Özlek [52], anemia, chronic kidney disease, and iron deficiency were independently associated with mortality from any cause.

The study group of patients was hospitalized during the COVID-19 pandemic, during which a significant impact of the pandemic on dietary habits and physical activity levels was observed [53]. Lockdown-related restrictions and reduced availability of medical care may have contributed to poorer diet quality, exacerbating malnutrition and impairing patients’ overall condition [54]. In addition, a lack of regular physical activity exacerbated the loss of muscle mass and led to greater progression of heart failure [55]. The research highlights the need to implement nutritional and rehabilitation strategies, especially in crises, to prevent negative health outcomes in this patient group [56,57,58]. Currently, based on an increasing number of articles, there is a growing interest in the nutritional status of patients in the medical literature. Findings suggest that there is an urgent need for preoperative and pre-hospitalization nutritional interventions, which can contribute to reducing the length of hospitalization, reducing the incidence of complications and lowering mortality [59]. For this reason, it seems optimal to assess nutritional status with appropriate treatment in the pre-hospitalization period or to introduce nutritional assessment in out-of-hospital treatment to identify and undertake treatment prior to hospitalization. In addition, in-hospital nutritional treatment should be intensified, especially in patients who are expected to have a prolonged period of hospitalization.

### 4.1. Study Limitations

The test has several limitations. One is the use of albumin levels as an indicator of nutritional status, which can be modified by factors not directly related to malnutrition, such as inflammation and hydration. In addition, the study did not include a control group of patients without heart failure, which limits the ability to clearly determine the specific impact of malnutrition on clinical outcomes in this population. Due to the retrospective design, data analysis was limited to information available in medical records, which may have affected the completeness and accuracy of the results. Another limitation of the study was the lack of inclusion of body composition assessment methods, such as bioelectrical impedance analysis (BIA) or muscle mass measurements, which could have provided more detailed information on malnutrition. In addition, the analysis included patients from a single center, which may limit the generalizability of the results to the broader population of heart failure patients. In addition, the majority of participants were elderly, which may not reflect the specific nutritional challenges in younger patients. Although the number of patients in the study was relatively large, it may still be insufficient to fully assess all variables affecting nutritional status and clinical outcomes.

### 4.2. Practical Implications

Future studies should consider additional methods of assessing malnutrition, such as body composition analysis and muscle mass measurement, to gain a more accurate picture of nutritional deficits in patients with heart failure. In addition, the use of biochemical markers, such as prealbumin or transferrin, may improve the predictive value of assessing nutritional status to clinical outcomes. Given the limitations of the single-center nature of the analysis, future studies should include a larger and more diverse patient population, taking into account data from different clinical centers. Additionally, it would be worthwhile to explore differences in nutritional challenges between younger and older heart failure patients, which could influence dietary intervention strategies. Future studies should focus on analyzing subgroups based on the severity of HF or different nutritional states. To better understand the relationship between malnutrition and mortality, intervention studies are needed in which targeted dietary strategies are implemented and their effects on patient outcomes are monitored over the long term.

Given that albumin levels can be modified by inflammation and hydration, future studies should include analysis of inflammatory markers (e.g., CRP) to better understand the interaction between nutritional status and the course of heart failure. Future research should focus on the impact of post-hospitalization nutritional strategies on the long-term prognosis of patients with heart failure. Monitoring patients after discharge could provide valuable data on the effectiveness of nutritional rehabilitation in improving quality of life and reducing the risk of readmissions. It is particularly important to identify patients at high risk of nutrition, allowing targeted interventions to be implemented, improving patient outcomes and reducing the burden on health systems.

## 5. Conclusions

Abnormal nutritional status among hospitalized heart failure patients is associated with longer hospitalization duration and higher mortality. In addition to clinical factors, the CHILD-PUGH scale can help estimate the length of hospitalization of patients with heart failure. It is necessary to determine the impact of nutritional status on the outcome of patients with heart failure in further multicenter prospectives or interventional studies.

## Figures and Tables

**Table 1:** Standardized research instruments used in the study.

Research Tool	Clinical Use	Structure	Scoring	Interpretation
CHILD-PUGH [26]	Assessment of liver disease severity and prognosis in patients with chronic liver disease.	Five parameters: bilirubin, albumin, INR, ascites, and hepatic encephalopathy levels.	5–15 points	A higher score indicates greater disease severity and a worse prognosis for the patient.
The Model for End-Stage Liver Disease (MELD) [27]	Assessment of the severity of liver disease and indications for liver transplantation.	Three parameters: creatinine, bilirubin and INR levels.	6–40 points,	A higher score indicates greater disease severity and a higher 3-month mortality rate.
Nutritional Risk Screening 2002 (NRS 2002) [28]	Nutritional risk assessment of hospitalized patients.	Three parameters: patient age, eating disorder, and severity of illness.	0 to 7 points	A score of ≥3 indicates increased nutritional risk, while when a patient scores <3, another assessment should be conducted within a week.

**Table 2:** Clinical characteristics of 213 cardiac patients.

Variable		Statistics
HGB (g/dL):	M ± SD	12.0 ± 6.4
	Me [Q1; Q3]	11.5 [10.1; 12.8]
	Min–Max	3.1–99.0
MCV (fl):	M ± SD	87.3 ± 9.5
	Me [Q1; Q3]	87.6 [82.8; 92.8]
	Min–Max	55.2–120.0
WBC (×10^9^/L):	M ± SD	8.1 ± 3.2
	Me [Q1; Q3]	7.2 [6.0; 9.4]
	Min–Max	2.3–22.9
PLT (×10^3^/μL):	M ± SD	229 ± 137
	Me [Q1; Q3]	203 [153; 284]
	Min–Max	59–1546
CRP (mg/dL):	M ± SD	19.1 ± 29.8
	Me [Q1; Q3]	19.2 [3.4; 19.6]
	Min–Max	0.1–240.2
ALT (U/L):	M ± SD	35.9 ± 49.0
	Me [Q1; Q3]	23 [15; 34]
	Min–Max	4–476
AST (U/L):	M ± SD	41.5 ± 59.4
	Me [Q1; Q3]	28 [21; 41]
	Min–Max	10–689
Creatinine (mg/dL):	M ± SD	1.60 ± 0.89
	Me [Q1; Q3]	1.36 [1.11; 1.83]
	Min–Max	0.62–7.74
Ferrum (ng/mL):	M ± SD	44.7 ± 27.1
	Me [Q1; Q3]	36 [27; 56]
	Min–Max	10–166
Albumin (g/dL):	M ± SD	3.63 ± 0.46
	Me [Q1; Q3]	3.7 [3.4; 3.9]
	Min–Max	1.8–4.8
CHILD-PUGH (score)	M ± SD	6.0 ± 1.3
	Me [Q1; Q3]	6 [5; 7]
	Min–Max	5–10
MELD (score)	M ± SD	24.5 ± 4.5
	Me [Q1; Q3]	24 [21; 28]
	Min–Max	13–40
Bilirubin (mg/dL):	M ± SD	1.35 ± 1.34
	Me [Q1; Q3]	0.90 [0.75; 1.50]
	Min–Max	0.23–14.30
PT/INR:	M ± SD	1.69 ± 1.02
	Me [Q1; Q3]	1.36 [1.17; 1.70]
	Min–Max	0.90–8.66
%SATTF:	M ± SD	14.4 ± 9.8
	Me [Q1; Q3]	12.0 [8.4; 17.4]
	Min–Max	3.1–8.0
Ferritin (μg/L):	M ± SD	164 ± 247
	Me [Q1; Q3]	95 [40; 204]
	Min–Max	5.5–2000
Ferritin (mg/L):	M ± SD	164 ± 247
	Me [Q1; Q3]	95 [40; 204]
	Min–Max	5.5–2000
APTT (s):	M ± SD	38.4 ± 10.9
	Me [Q1; Q3]	35.8 [31.2; 42.0]
	Min–Max	23.2–99.3

BMI: Body Mass Index; Me: Median; Q1: First quartile; Q3: Third quartile; M: Mean; SD: Standard deviation; %SATTF: Percentage of transferrin saturation; MELD: Model for End-Stage Liver Disease; HGB: Hemoglobin; MCV: Mean Corpuscular Volume; WBC: White Blood Cells; PLT: Platelets; CRP: C-Reactive Protein; ALT: Alanine aminotransferase; AST: Aspartate aminotransferase; PT/INR: Prothrombin Time/International Normalized Ratio; APTT: Activated Partial Thromboplastin Time.

**Table 3:** Clinical characteristics of 213 cardiac patients in groups differing in nutritional status.

Variable		Nutritional Status		*p* Value
		<3 (*N* = 181)	≥3 (*N* = 32)	
Male	*N* (%)	134 (74.0)	19 (59.4)	0.134
Age (years)	Me [Q1; Q3]	75 [66; 84]	85 [79; 91]	<0.001
Body height (cm)	Me [Q1; Q3]	170 [165; 176]	167 [156; 173]	0.005
Body mass (kg)	Me [Q1; Q3]	80 [70; 93]	75 [62; 80]	0.007
BMI (kg/m^2^)	Me [Q1; Q3]	27.4 [24.1; 31.3]	25.8 [24.0; 28.5]	0.148
Treatment effect:				0.018
Cured	N (%)	172 (95.0)	27 (84.4)	
Further treatment	N (%)	5 (2.8)	1 (3.1)	
Death	N (%)	4 (2.2)	4 (12.5)	
Hospitalization time (days)	Me [Q1; Q3]	9 [6; 15]	12 [9; 19]	0.027
HGB (g/dL)	Me [Q1; Q3]	11.7 [10.3; 13.0]	10.5 [9.4; 11.2]	0.001
MCV (fl)	Me [Q1; Q3]	87.7 [82.8; 92.1]	87.4 [82.4; 96.3]	0.556
WBC (×10^9^/L)	Me [Q1; Q3]	7.4 [6.0; 9.2]	6.9 [6.0; 10.6]	0.797
PLT (×10^3^/μL)	Me [Q1; Q3]	193 [152; 269]	225 [177; 293]	0.126
CRP (mg/dL)	Me [Q1; Q3]	8.6 [3.2; 17.7]	16.6 [3.7; 47.4]	0.053
ALT (U/L)	Me [Q1; Q3]	23 [15; 33]	23 [17; 37]	0.558
AST (U/L)	Me [Q1; Q3]	28 [21; 40]	31 [22; 45]	0.377
Creatinine (mg/dL)	Me [Q1; Q3]	1.36 [1.11; 1.83]	1.37 [1.07; 1.76]	0.908
Ferrum (ng/mL)	Me [Q1; Q3]	39 [28; 57]	32 [20; 42]	0.009
Albumin (g/dL)	Me [Q1; Q3]	3.70 [3.41; 3.87]	3.60 [3.15; 3.83]	0.138
CHILD-PUGH	Me [Q1; Q3]	6 [5; 7]	5 [5; 6]	0.572
MELD	Me [Q1; Q3]	24 [21; 28]	23 [20; 28]	0.646
Bilirubin	Me [Q1; Q3]	0.93 [0.75; 1.64]	0.88 [0.72; 1.20]	0.263
PT/INR	Me [Q1; Q3]	1.37 [1.17; 1.70]	1.22 [1.16; 1.62]	0.305
%SATTF	Me [Q1; Q3]	12.3 [8.5; 17.4]	9.5 [6.5; 17.9]	0.270
Ferritin (μg/L)	Me [Q1; Q3]	86 [39; 199]	131 [56; 240]	0.100
TIBC	Me [Q1; Q3]	336 [283; 402]	292 [227; 373]	0.012
APTT	Me [Q1; Q3]	36.5 [31.2; 42.1]	34.8 [30.8; 41.6]	0.597

BMI: Body Mass Index; N: Number of observations; Me: Median; Q1: First quartile; Q3: Third quartile; TIBC: Total Iron-Binding Capacity; %SATTF: Percentage of transferrin saturation; MELD: Model for End-Stage Liver Disease; HGB: Hemoglobin; MCV: Mean Corpuscular Volume; WBC: White Blood Cells; PLT: Platelets; CRP: C-Reactive Protein; ALT: Alanine aminotransferase; AST: Aspartate aminotransferase; PT/INR: Prothrombin Time/International Normalized Ratio; APTT: Activated Partial Thromboplastin Time.

**Table 4:** Clinical characteristics of 213 cardiac patients in groups differing in length of hospitalization.

Variable		Duration of Hospitalization (days)			*p* Value
		Up to 7 Days *N* = 73	8 to 13 Days *N* = 74	14 and over *N* = 66	
Male	*N* (%)	49 (67.1)	52 (70.3)	52 (78.8)	0.291
Age (years)	Me [Q1; Q3]	78 [65; 86]	76 [70; 84]	76 [66; 84]	0.854
Body height	Me [Q1; Q3]	170 [164; 176]	171 [160; 176]	170 [166; 175]	0.991
Body mass	Me [Q1; Q3]	74 [62; 83]	82 [73; 95]	81 [72; 96]	0.001
BMI (kg/m^2^)	Me [Q1; Q3]	25.2 [22.1; 29.4]	28.4 [25.0; 31.5]	27.6 [25.2; 33.7]	<0.001
Nutritional assessment (NRS)	Me [Q1; Q3]	1 [0; 1]	1 [1; 1]	1 [0; 2]	0.570
Treatment effect:					0.007
Cured	N (%)	71 (97.2)	72 (97.3)	56 (84.9)	
further treatment	N (%)	1 (1.4)	2 (2.7)	3 (4.5)	
Death	N (%)	1 (1.4)	0 (0.0)	7 (10.6)	
HGB (g/dL)	Me [Q1; Q3]	11.5 [10.3; 13.1]	11.5 [9.9; 12.7]	11.6 [10.1; 12.9]	0.892
MCV (fl)	Me [Q1; Q3]	87.6 [81.3; 92.1]	88.5 [81.5; 92.3]	87.2 [84.1; 94.0]	0.683
WBC (×10^9^/L)	Me [Q1; Q3]	7.0 [6.1; 8.9]	7.1 [5.9; 8.9]	8.0 [6.0; 10.4]	0.322
PLT (×10^3^/μL)	Me [Q1; Q3]	204 [153; 268]	189 [153; 286]	220 [149; 289]	0.860
CRP (mg/dL)	Me [Q1; Q3]	6.7 [2.4; 13.5]	7.0 [3.1; 16.7]	17.0 [7.2; 23.4]	0.001
ALT (U/L)	Me [Q1; Q3]	22 [15; 29]	26 [16; 37]	23 [16; 32]	0.391
AST (U/L)	Me [Q1; Q3]	27 [21; 36]	29 [22; 42]	30 [23; 42]	0.182
Creatinine (mg/dL)	Me [Q1; Q3]	1.25 [1.00; 1.67]	1.37 [1.08; 1.83]	1.55 [1.22; 1.85]	0.019
Ferrum (ng/mL)	Me [Q1; Q3]	42 [31; 58]	39 [28; 57]	29 [21; 46]	<0.001
Albumin (g/dL)	Me [Q1; Q3]	3.79 [3.57; 3.99]	3.70 [3.51; 3.86]	3.50 [3.20; 3.70]	<0.001
CHILD-PUGH	Me [Q1; Q3]	5 [7; 7]	5 [5; 6]	6 [5; 7]	0.023
MELD	Me [Q1; Q3]	24 [21; 27]	24 [21; 27]	24 [21; 29]	0.512
Bilirubin	Me [Q1; Q3]	0.89 [0.75; 1.49]	0.89 [0.69; 1.41]	0.98 [0.74; 1.89]	0.327
PT/INR	Me [Q1; Q3]	1.34 [1.16; 1.78]	1.34 [1.11; 1.66]	1.38 [1.20; 1.75]	0.449
%SATTF	Me [Q1; Q3]	12.9 [9.1; 17.4]	12.1 [8.4; 17.8]	10.9 [7.0; 17.0]	0.228
Ferritin	Me [Q1; Q3]	42 [31; 58]	39 [28; 57]	29 [21; 46]	<0.001
TIBC	Me [Q1; Q3]	338 [293; 420]	337 [288; 402]	304 [249; 376]	0.024
APTT	Me [Q1; Q3]	35.6 [32.0; 41.2]	37.4 [29.9; 42.2]	35.4 [31.9; 41.4]	0.996

BMI: Body Mass Index; N: Number of observations; Me: Median; Q1: First quartile; Q3: Third quartile; TIBC: Total Iron-Binding Capacity; %SATTF: Percentage of transferrin saturation; MELD: Model for End-Stage Liver Disease; HGB: Hemoglobin; MCV: Mean Corpuscular Volume; WBC: White Blood Cells; PLT: Platelets; CRP: C-Reactive Protein; ALT: Alanine aminotransferase; AST: Aspartate aminotransferase; PT/INR: Prothrombin Time/International Normalized Ratio; APTT: Activated Partial Thromboplastin Time.

**Table 5:** Clinical characteristics of 213 cardiac patients in treatment effect groups and results of significance tests.

Variable		Group of Patients			*p*-Value
		A (*N* = 199)	B (*N* = 6)	C (*N* = 8)	
Male	*N* (%)	142 (71.4)	4 (66.7)	7 (87.5)	0.585
Age (years)	Me [Q1; Q3]	76 [66; 85]	76 [67; 81]	84 [78; 85]	0.349
Body height	Me [Q1; Q3]	170 [164; 176]	171 [160; 176]	170 [166; 175]	0.962
Body mass	Me [Q1; Q3]	80 [68; 92]	78 [70; 80]	80 [71; 92]	0.867
BMI (kg/m^2^)	Me [Q1; Q3]	27.2 [24.0; 31.2]	26.0 [24.5; 30.1]	27.5 [26.4; 30.7]	0.868
Nutritional assessment (NRS)	Me [Q1; Q3]	1 [0; 1]	1 [1; 2]	2.5 [1.5; 5]	<0.001
Hospitalization time (days)	Me [Q1; Q3]	9 [6; 15]	18 [10; 32]	25 [20; 39]	<0.001
HGB (g/dL)	Me [Q1; Q3]	11.5 [10.1; 12.8]	12.1 [9.8; 15.4]	10.4 [9.9; 12.2]	0.453
MCV (fl)	Me [Q1; Q3]	87.6 [82.9; 92.9]	90.5 [78.4; 96.1]	83.2 [81.5; 89.4]	0.559
WBC (×10^9^/L)	Me [Q1; Q3]	7.2 [6.0; 9.2]	8.9 [6.6; 10.7]	6.6 [5.8; 11.2]	0.626
PLT (×10^3^/μL)	Me [Q1; Q3]	199 [153; 279]	230 [156; 369]	218 [154; 310]	0.835
CRP (mg/dL)	Me [Q1; Q3]	9.0 [3.2; 19.2]	7.7 [6.7; 19.8]	21.5 [7.8; 106.3]	0.129
ALT (U/L)	Me [Q1; Q3]	23 [15; 34]	34 [28; 40]	14 [11; 24]	0.019
AST (U/L)	Me [Q1; Q3]	28 [21; 41]	42 [29; 51]	24 [22; 27]	0.117
Creatinine (mg/dL)	Me [Q1; Q3]	1.35 [1.10; 1.83]	1.45 [1.06; 2.76]	1.69 [1.31; 2.09]	0.368
Ferrum (ng/mL)	Me [Q1; Q3]	38 [27; 57]	32 [29; 34]	25 [17; 34]	0.059
Albumin (g/dL)	Me [Q1; Q3]	3.70 [3.41; 3.90]	3.31 [3.00; 3.60]	3.17 [2.80; 3.45]	<0.001
CHILD-PUGH	Me [Q1; Q3]	5 [5; 7]	7 [6; 9]	6 [6; 8]	0.070
MELD	Me [Q1; Q3]	24 [21; 27]	28 [22; 29]	25 [22; 29]	0.529
Bilirubin	Me [Q1; Q3]	0.89 [0.75; 1.47]	2.57 [0.68; 3.35]	1.53 [0.61; 1.83]	0.343
PT/INR	Me [Q1; Q3]	1.35 [1.16; 1.69]	2.12 [1.22; 3.57]	1.52 [1.17; 2.04]	0.336
%SATTF	Me [Q1; Q3]	12.0 [8.5; 17.4]	11.4 [8.0; 15.6]	9.7 [6.5; 15.1]	0.585
Ferritin	Me [Q1; Q3]	38 [27; 57]	32 [29; 34]	25 [17; 34]	0.059
TIBC	Me [Q1; Q3]	336 [282; 401]	252 [218; 338]	241 [215; 353]	0.035
APTT	Me [Q1; Q3]	35.7 [30.8; 42.2]	36.9 [28.5; 40.8]	37.0 [33.8; 39.6]	0.904

Group A—completion of the therapeutic process; Group B—referral for further treatment in another hospital or outpatient treatment; Group C—death; BMI: Body Mass Index; N: Number of observations; Me: Median; Q1: First quartile; Q3: Third quartile; TIBC: Total Iron-Binding Capacity; %SATTF: Percentage of transferrin saturation; MELD: Model for End-Stage Liver Disease; HGB: Hemoglobin; MCV: Mean Corpuscular Volume; WBC: White Blood Cells; PLT: Platelets; CRP: C-Reactive Protein; ALT: Alanine aminotransferase; AST: Aspartate aminotransferase; PT/INR: Prothrombin Time/International Normalized Ratio; APTT: Activated Partial Thromboplastin Time.

## Data Availability

The data will be available by contacting the corresponding author due to privacy, legal and ethical reasons.

## References

[1] Savarese G., Lund L.H. (2017). Global Public Health Burden of Heart Failure. Card. Fail. Rev..

[2] GBD (2018). 2017 Disease and Injury Incidence and Prevalence Collaborators Global, Regional, and National Incidence, Prevalence, and Years Lived with Disability for 354 Diseases and Injuries for 195 Countries and Territories, 1990–2017: A Systematic Analysis for the Global Burden of Disease Study 2017. Lancet.

[3] Christiansen M.N., Køber L., Weeke P., Vasan R.S., Jeppesen J.L., Smith J.G., Gislason G.H., Torp-Pedersen C., Andersson C. (2017). Age-Specific Trends in Incidence, Mortality, and Comorbidities of Heart Failure in Denmark, 1995 to 2012. Circulation.

[4] Tsao C.W., Lyass A., Enserro D., Larson M.G., Ho J.E., Kizer J.R., Gottdiener J.S., Psaty B.M., Vasan R.S. (2018). Temporal Trends in the Incidence of and Mortality Associated With Heart Failure With Preserved and Reduced Ejection Fraction. JACC Heart Fail..

[5] Smeets M., Vaes B., Mamouris P., Van Den Akker M., Van Pottelbergh G., Goderis G., Janssens S., Aertgeerts B., Henrard S. (2019). Burden of Heart Failure in Flemish General Practices: A Registry-Based Study in the Intego Database. BMJ Open.

[6] Conrad N., Judge A., Tran J., Mohseni H., Hedgecott D., Crespillo A.P., Allison M., Hemingway H., Cleland J.G., McMurray J.J.V. (2018). Temporal Trends and Patterns in Heart Failure Incidence: A Population-Based Study of 4 Million Individuals. Lancet.

[7] (2023). Towarzystwa Kardiologicznego Niewydolność Serca w Polsce; Asocjacja Niewydolności Serca Polskiego. https://www.niewydolnosc-serca.pl/doc/ANS_raport_01.09_.pdf.

[8] Zaleska-Kociecka M., Celinska-Spodar M., Podwojcic K., Maluchnik M., Walkiewicz D., Hryniewiecki T., Leszek P. (2022). COVID-19 Pandemic Impact on Heart Failure Epidemiology and Outcomes in Poland. National Database Study. Eur. Heart J..

[9] Lv J., Zhang L., Yixi Z., Zhang Y., Li X., Yang C., Wang M. (2024). The Global Burden of Cardiovascular Disease Attributable to Diet Low in Fiber among People Aged 60 Years and Older, 1990–2019: An Age–Period–Cohort Analysis of the Global Burden of Disease Study. BMC Public Health.

[10] Park J.H., Moon J.H., Kim H.J., Kong M.H., Oh Y.H. (2020). Sedentary Lifestyle: Overview of Updated Evidence of Potential Health Risks. Korean J. Fam. Med..

[11] Liu W., Dostdar-Rozbahani A., Tadayon-Zadeh F., Akbarpour-Beni M., Pourkiani M., Sadat-Razavi F., Barfi V., Shahedi V. (2022). Insufficient Level of Physical Activity and Its Effect on Health Costs in Low- and Middle-Income Countries. Front. Public Health.

[12] Ghodeshwar G.K., Dube A., Khobragade D. (2023). Impact of Lifestyle Modifications on Cardiovascular Health: A Narrative Review. Cureus.

[13] Yeates K., Lohfeld L., Sleeth J., Morales F., Rajkotia Y., Ogedegbe O. (2015). A Global Perspective on Cardiovascular Disease in Vulnerable Populations. Can. J. Cardiol..

[14] Martínez-González M.A., Gea A., Ruiz-Canela M. (2019). The Mediterranean Diet and Cardiovascular Health. Circ. Res..

[15] Sandri E., Sguanci M., Cantín Larumbe E., Cerdá Olmedo G., Werner L.U., Piredda M., Mancin S. (2024). Plant-Based Diets versus the Mediterranean Dietary Pattern and Their Socio-Demographic Determinants in the Spanish Population: Influence on Health and Lifestyle Habits. Nutrients.

[16] Sam-Yellowe T.Y. (2024). Nutritional Barriers to the Adherence to the Mediterranean Diet in Non-Mediterranean Populations. Foods.

[17] McDonagh T.A., Metra M., Adamo M., Gardner R.S., Baumbach A., Böhm M., Burri H., Butler J., Čelutkienė J., Chioncel O. (2021). 2021 ESC Guidelines for the Diagnosis and Treatment of Acute and Chronic Heart Failure. Eur. Heart J..

[18] Ojo O., Adegboye A.R.A. (2023). The Effects of Nutrition on Chronic Conditions. Nutrients.

[19] Bansal N., Alharbi A., Shah M., Altorok I., Assaly R., Altorok N. (2024). Impact of Malnutrition on the Outcomes in Patients Admitted with Heart Failure. J. Clin. Med..

[20] Abdoul Carime N., Cottenet J., Clerfond G., Eschalier R., Quilliot D., Eicher J.-C., Joly B., Quantin C. (2022). Impact of Nutritional Status on Heart Failure Mortality: A Retrospective Cohort Study. Nutr. J..

[21] Weidenhammer A., Prausmueller S., Spinka G., Goliasch G., Arfsten H., Pavo N., Huelsmann M., Bartko P. (2022). Malnutrition in Patients with Chronic Heart Failure. Eur. Heart J..

[22] Murphy S.P., Kakkar R., McCarthy C.P., Januzzi J.L. (2020). Inflammation in Heart Failure: JACC State-of-the-Art Review. J. Am. Coll. Cardiol..

[23] Xu D., Lu Y., Wang Y., Li F. (2024). The Obesity Paradox and 90 Day Mortality in Chronic Critically Ill Patients: A Cohort Study Using a Large Clinical Database. Eur. J. Med. Res..

[24] Screever E.M., van der Wal M.H.L., van Veldhuisen D.J., Jaarsma T., Koops A., van Dijk K.S., Warink-Riemersma J., Coster J.E., Westenbrink B.D., van der Meer P. (2023). Comorbidities Complicating Heart Failure: Changes over the Last 15 Years. Clin. Res. Cardiol..

[25] Kałużna-Oleksy M., Krysztofiak H., Migaj J., Wleklik M., Dudek M., Uchmanowicz I., Lesiak M., Straburzyńska-Migaj E. (2020). Relationship between Nutritional Status and Clinical and Biochemical Parameters in Hospitalized Patients with Heart Failure with Reduced Ejection Fraction, with 1-Year Follow-Up. Nutrients.

[26] Pugh R.N., Murray-Lyon I.M., Dawson J.L., Pietroni M.C., Williams R. (1973). Transection of the Oesophagus for Bleeding Oesophageal Varices. Br. J. Surg..

[27] Malinchoc M., Kamath P.S., Gordon F.D., Peine C.J., Rank J., ter Borg P.C.J. (2000). A Model to Predict Poor Survival in Patients Undergoing Transjugular Intrahepatic Portosystemic Shunts. Hepatology.

[28] Kondrup J., Rasmussen H.H., Hamberg O., Stanga Z. (2003). Ad Hoc ESPEN Working Group Nutritional Risk Screening (NRS 2002): A New Method Based on an Analysis of Controlled Clinical Trials. Clin. Nutr..

[29] R Core Team (2024). R: A Language and Environment for Statistical Computing.

[30] (2014). Global Status Report on Noncommunicable Diseases. https://www.who.int/publications/i/item/9789241564854.

[31] Projections of Global Mortality and Burden of Disease from 2002 to 2030|PLOS Medicine. https://journals.plos.org/plosmedicine/article?id=10.1371/journal.pmed.0030442.

[32] Tyrovola D., Soulaidopoulos S., Tsioufis C., Lazaros G. (2023). The Role of Nutrition in Cardiovascular Disease: Current Concepts and Trends. Nutrients.

[33] Chiuve S.E., McCullough M.L., Sacks F.M., Rimm E.B. (2006). Healthy Lifestyle Factors in the Primary Prevention of Coronary Heart Disease among Men: Benefits among Users and Nonusers of Lipid-Lowering and Antihypertensive Medications. Circulation.

[34] van Trier T.J., Mohammadnia N., Snaterse M., Peters R.J.G., Jørstad H.T., Bax W.A. (2022). Lifestyle Management to Prevent Atherosclerotic Cardiovascular Disease: Evidence and Challenges. Neth. Heart J..

[35] Andreadi A., Bellia A., Di Daniele N., Meloni M., Lauro R., Della-Morte D., Lauro D. (2022). The Molecular Link between Oxidative Stress, Insulin Resistance, and Type 2 Diabetes: A Target for New Therapies against Cardiovascular Diseases. Curr. Opin. Pharmacol..

[36] Kosmas C.E., Bousvarou M.D., Kostara C.E., Papakonstantinou E.J., Salamou E., Guzman E. (2023). Insulin Resistance and Cardiovascular Disease. J. Int. Med. Res..

[37] Caturano A., Rocco M., Tagliaferri G., Piacevole A., Nilo D., Di Lorenzo G., Iadicicco I., Donnarumma M., Galiero R., Acierno C. (2025). Oxidative Stress and Cardiovascular Complications in Type 2 Diabetes: From Pathophysiology to Lifestyle Modifications. Antioxidants.

[38] Li Y., Liu Y., Liu S., Gao M., Wang W., Chen K., Huang L., Liu Y. (2023). Diabetic Vascular Diseases: Molecular Mechanisms and Therapeutic Strategies. Sig Transduct. Target. Ther..

[39] Elendu C., Amaechi D.C., Elendu T.C., Ashna M., Ross-Comptis J., Ansong S.O., Egbunu E.O., Okafor G.C., Jingwa K.A., Akintunde A.A. (2023). Heart Failure and Diabetes: Understanding the Bidirectional Relationship. Medicine.

[40] Gajewska D., Lange E., Kęszycka P., Białek-Dratwa A., Gudej S., Świąder K., Giermaziak W., Kostelecki G., Kret M., Marlicz W. (2024). Recommendations on Dietary Treatment of Obesity in Adults: 2024 Position of the Polish Society of Dietetics. J. Health Inequal..

[41] Kobylińska M., Antosik K., Decyk A., Kurowska K. (2021). Malnutrition in Obesity: Is It Possible?. Obes. Facts.

[42] Kahokehr A.A., Sammour T., Wang K., Sahakian V., Plank L.D., Hill A.G. (2010). Prevalence of Malnutrition on Admission to Hospital—Acute and Elective General Surgical Patients. e-SPEN Eur. E-J. Clin. Nutr. Metab..

[43] Kutnik P., Wichowska O., Sysiak-Sławecka J., Szczukocka M., Rypulak E., Piwowarczyk P., Borys M., Czuczwar M. (2023). Malnutrition Risk in Elective Surgery Patients and Effectiveness of Preoperative Nutritional Interventions at a Pre-Anaesthetic Clinic: A 4-Year Apart, Single-Centre, Observational Study. Anaesthesiol. Intensive Ther..

[44] de Moraes A.C.O., da Fonseca-Neto O.C.L. (2018). The use of meld score (model for end-stage liver disease) and derivatives in cardiac transplantation. Arq. Bras. Cir. Dig..

[45] Wang Y., Veltkamp D.M.J., van der Boog P.J.M., Hemmelder M.H., Dekker F.W., de Vries A.P.J., Meuleman Y. (2022). Illness Perceptions and Medication Nonadherence to Immunosuppressants After Successful Kidney Transplantation: A Cross-Sectional Study. Transpl. Int..

[46] Naghashzadeh F., Noorali S., Hosseini-Baharanchi F.S., Shafaghi S., Sharif-Kashani B., Ahmadi Z.H., Keshmiri M.S. (2021). Comparison of Scores for Child-Pugh Criteria and Standard and Modified Models for End-Stage Liver Disease to Assess Cardiac Hepatopathy in Heart Transplant Recipients. Exp. Clin. Transpl..

[47] Doctoroff L., Herzig S.J. (2020). Predicting Patients at Risk for Prolonged Hospital Stays. Med. Care.

[48] Beck A.M., Knudsen A.W., Østergaard T.B., Rasmussen H.H., Munk T. (2021). Poor Performance in Nutrition Risk Screening May Have Serious Consequences for Hospitalized Patients. Clin. Nutr. ESPEN.

[49] Orell H., Pohju A., Tuokkola J., Junttila K., Heikkilä A., Österlund P., Schwab U., Mäkitie A. (2023). Time to Act!—A Cross-Sectional Study on How Nutritional Risk Increases during Hospitalization and Associates with Worse Outcome. Clin. Nutr. ESPEN.

[50] Pan D., Chen H. (2024). Relationship between Serum Albumin Level and Hospitalization Duration Following Percutaneous Coronary Intervention for Acute Coronary Syndrome. Sci. Rep..

[51] Ancion A., Allepaerts S., Robinet S., Oury C., Pierard L.A., Lancellotti P. (2019). Serum Albumin Level and Long-Term Outcome in Acute Heart Failure. Acta Cardiol..

[52] Köseoğlu F.D., Özlek B. (2024). Anemia and Iron Deficiency Predict All-Cause Mortality in Patients with Heart Failure and Preserved Ejection Fraction: 6-Year Follow-Up Study. Diagnostics.

[53] Bennett G., Young E., Butler I., Coe S. (2021). The Impact of Lockdown During the COVID-19 Outbreak on Dietary Habits in Various Population Groups: A Scoping Review. Front. Nutr..

[54] Laddu D.R., Biggs E., Kaar J., Khadanga S., Alman R., Arena R. (2023). The Impact of the COVID-19 Pandemic on Cardiovascular Health Behaviors and Risk Factors: A New Troubling Normal That May Be Here to Stay. Prog. Cardiovasc. Dis..

[55] Albert N.M., Sumser M., Bena J.F., Morrison S.L., Siegmund L.A., Reay R. (2024). Self-Perceptions Of Lifestyle Changes During Covid-19, Adherence To Heart Failure Self-Care Behaviors And Their Association With Heart Failure-Related Quality Of Life. J. Card. Fail..

[56] Cangelosi G., Grappasonni I., Pantanetti P., Scuri S., Garda G., Cuc Thi Thu N., Petrelli F. (2022). Nurse Case Manager Lifestyle Medicine (NCMLM) in the Type Two Diabetes Patient Concerning Post COVID-19 Pandemic Management: Integrated-Scoping Literature Review. Annali di Igiene Medicina Preventiva e di Comunità.

[57] Driggin E., Cohen L.P., Gallagher D., Karmally W., Maddox T., Hummel S.L., Carbone S., Maurer M.S. (2022). Nutrition Assessment and Dietary Interventions in Heart Failure: JACC Review Topic of the Week. J. Am. Coll. Cardiol..

[58] Lee H., Jeong S.Y., Choi H.R., Kang S.-M. (2021). Nutrition Intervention Process for Heart Failure Patients According to Their Nutritional Problems. Clin. Nutr. Res..

[59] Singer P., Blaser A.R., Berger M.M., Calder P.C., Casaer M., Hiesmayr M., Mayer K., Montejo-Gonzalez J.C., Pichard C., Preiser J.-C. (2023). ESPEN Practical and Partially Revised Guideline: Clinical Nutrition in the Intensive Care Unit. Clin. Nutr..

